# Enhancing Energy Absorption in PLA Prints Through Post-Thermal Treatment

**DOI:** 10.3390/polym18091138

**Published:** 2026-05-06

**Authors:** Roberto Orduz, Brayan Murgas, Sergio G. Torres-Cedillo, Jacinto Cortés-Pérez, Moises Jimenez-Martinez

**Affiliations:** 1 Tecnologico de Monterrey, School of Engineering and Sciences, Via Atlixcayotl 5718, Puebla 72453, Mexico; a01769661@tec.mx; 2 X Computational Physics Division, Los Alamos National Laboratory, Los Alamos, NM 87545, USA; bmurgas@lanl.gov; 3 Centro Tecnológico Aragón FES Aragón, Universidad Nacional Autónoma de México, Nezahualcóyotl 57171, Mexico; sergiotorres98@aragon.unam.mx (S.G.T.-C.); jacop@unam.mx (J.C.-P.)

**Keywords:** post-treatment, crushing, additive manufacturing, annealing, energy absorption

## Abstract

Energy absorption is a pivotal factor in impact mitigation, especially in engineering applications that demand lightweight and efficient mechanical absorbers. Traditionally, crash boxes are manufactured using metallic materials; however, advancements in additive manufacturing present new opportunities for producing lightweight prototypes quickly, without the need for specialized tooling. This study explores the potential of 3D-printed polylactic acid (PLA) components with tailored stiffness to optimize energy absorption performance. By combining material properties, manufacturing parameters, and design strategies, varying mechanical strengths were achieved. Furthermore, the performance of the best combination was enhanced through post-processing via thermal treatment, resulting in improved crushing metrics. Experimental results revealed a 33% increase in energy absorption for honeycomb structures with variable thickness designs, demonstrating the benefits of tailored stiffness distribution. These findings highlight the promise of variable-thickness 3D-printed composites as highly effective and customizable energy absorbers, offering innovative solutions for micromobility and other engineering applications.

## 1. Introduction

The continuous advancement of the automotive and aerospace industries, alongside the shift in design paradigms and the implementation of approaches aimed at meeting operational safety requirements, currently demands innovative engineering solutions for various passive safety systems. These solutions leverage the latest technological developments in manufacturing processes. In this context, additive manufacturing has emerged as a promising and versatile tool in industrial research. However, ongoing efforts are focused on improving quality, reducing costs, and optimizing production times [1].

Among the diverse applications of additive manufacturing, passive safety systems based on energy absorption have gained significant relevance in the automotive, aerospace, and aeronautical industries, particularly in the performance analysis of these energy-absorbing devices [2,3]. In this context, various concepts have been explored, including bio-inspired geometries [4,5], thin-walled corrugated structures [6], lattice metamaterials [7], and others.

**Figure 1:**
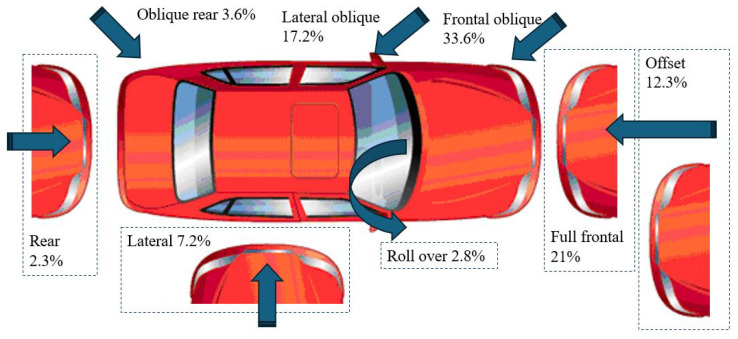
Percentage Distribution of Impacts on a Vehicle.

As a specific case study, the automotive industry is regulated by the National Highway Traffic Safety Administration, which mandates the strategic implementation of energy dissipators based on the most common types of collisions. Research in this field has primarily focused on various impact scenarios, including frontal impact, side impact [8], oblique impact and rollover [9]. Figure 1 summarizes the main types of crashes on vehicles where it can be seen that the frontal is the main type of crash [10].

**Figure 2:**
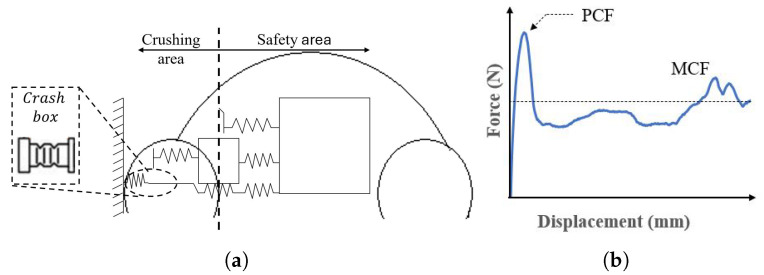
Crushing behaviour: (**a**) crash box location and (**b**) crushing response.

The deformation vehicle, illustrated in Figure 2a, consists of structural components designed to work synergistically to absorb and dissipate energy during an impact. A critical element within this system is the crash box, which is strategically positioned between the chassis and the front and rear bumpers. While traditionally manufactured from steel or aluminum, recent advancements have introduced innovative technologies, including advanced alloys [11], composite materials, and other cutting-edge solutions [12]. The mechanical response of the crushing element, determined by its displacement, is characterized by two key parameters: the Peak Crush Force (PCF) and the Mean Crushing Force (MCF), as depicted in Figure 2b.

In this context, the Crash Box functions as a passive safety device and plays a crucial role by being implemented to achieve total energy absorption at low speeds and programmed deformation. Its primary purpose is to dissipate energy, thereby reducing damage to internal components that are more complex to maintain, preserving the structural integrity of the vehicle [13].

These are specialized load cases designed to optimize energy absorption, with applications extending beyond mobility. In the context of transportation, these solutions are not only employed to safeguard critical components of vehicles but also play a crucial role in mitigating the severity of biomechanical injuries sustained by vehicle occupants and pedestrians during collisions. By dissipating impact forces effectively, these energy absorption systems reduce the likelihood of life-threatening injuries and improve overall safety. Moreover, their implementation is increasingly being explored in micromobility devices and lightweight vehicles, where compact and efficient designs are essential not only to improve the performance, also to reduce or mitigate the risk of injury by the transport users and pedestrian [14,15].

Additive manufacturing is a cornerstone of Industry 4.0 and has rapidly grown due to advantages such as accessibility, on-demand production, and rapid prototyping. Within this context, one of the most commonly used additive manufacturing processes is material extrusion [16], where a polymer filament is typically extruded to create the required geometries layer by layer. However, due to the layer-by-layer construction nature of printed parts, their mechanical properties are often reduced as a result of anisotropy caused by voids between layers [17].

Bambach et al. conducted an evaluation of crushing performance in hybrid metals reinforced with carbon fiber, demonstrating improvements in the mean crushing load and specific energy absorption [18]. This hybrid material presents a clear potential for industrial implementation due to its enhanced performance. On the other hand, the impact and energy absorption analysis of metals and polymers using foam fillers, as studied by [19], highlights advantages in controlling localized impacts. These findings, derived from numerical and experimental analyses, suggest possible applications in the aerospace, automotive, and civil engineering sectors.

Thanks to the flexibility in geometry enabled by additive manufacturing, an endless variety of possibilities arises. Among these, several notable studies stand out. For instance, the experimental analysis conducted by [20] explored PLA fillers with random shapes combined with thin metal walls. This study provides a process method that adapts meso-structural parameters to serve as a design guide for high-performance mechanical energy absorbers.

Adding to this, the study conducted by [21] focused on energy absorption in a honeycomb-type geometry. A calibrated PLA specimen was subjected to numerical and experimental analysis, demonstrating suitable behavior under quasi-static loads as an energy absorber when compared to aluminum and thermoplastic polymers. Similarly, the design and analysis of geometric relationships for a device aimed at crushing performance, as conducted by [22], utilized a multi-objective algorithm to maximize specific energy absorption (SEA). This study concluded with a methodological proposal for designing mechanical energy absorption structures with optimized objectives.

Complementing the previous discussion, studies on metamaterials have demonstrated that absorbers can be designed based on geometry with varying Poisson ratios and one or multiple plateau zones. These designs offer a comparative advantage due to programmed collapse, which can be tailored for specific design cases. In this context, the research presented by [23] evaluates a two-stage deformation geometry, integrating mechanical property enhancements that provide valuable references for applications in load-bearing and vibration analysis.

Additionally, auxetic geometries, such as the one evaluated by [24], examine and showcase the mechanical behavior of a proposed auxetic structure. Numerical predictions of the plateau stress zone achieved an error of less than 10% compared to experimental tests, demonstrating the reliability and precision of the approach.

The most common approach to enhancing mechanical performance is through design modifications. However, an alternative method involves applying post-thermal processes to improve mechanical strength. This enhancement can be achieved through specific thermal treatments. Thermal treatments have been extensively studied in metals, primarily to modify their microstructure and enhance mechanical, physical, chemical, or manufacturing properties through the application of heat [25]. However, to the best knowledge of the authors, research on the thermal treatment of polymers remains limited. This process has shown potential as a preparatory step in manufacturing, where it can homogenize the material and improve its tensile strength, as demonstrated in studies on PLA [26] and other polymers such as Onyx [27]. Furthermore, thermal treatment has been found to reduce porosity, although its parameters must be carefully defined to mitigate the risk of deformation [28]. Additionally, this technique contributes to minimizing the disparity in mechanical properties observed between extrusion-based 3D printing and injection molding processes [29].

This study analyzes various internal topologies of crash boxes fabricated using PLA extrusion, focusing on the correlation between design features and manufacturing process parameters. Additionally, it proposes the application of thermal treatment as a post-processing technique to enhance the energy absorption capacity of these structures. By combining geometry optimization and tailored material properties, the research aims to provide insights into improving the mechanical performance of lightweight mechanical energy absorbers.

## 2. Energy Absorption

According to statistics, frontal impacts in automobiles are responsible for more deaths and severe injuries than any other type of accident (Figure 1). In this context, the worst-case scenario during a collision occurs when the entire energy transformation is absorbed by a single vehicle. Considering the mathematical idealization of this specific case, the representation is given by Equation (1), where $S_{f}$ represents the total deformation, *F* is the impact force, *m* is the vehicle’s mass, and $v_{0}$ is the initial velocity before the frontal collision.

(1) $$\int_{0}^{S_{f}}Fds=\frac{1}{2}m\nu_{o}^{2}$$

Similarly, assuming that the impact force is constant, we could use the momentum conservation equation (Equation (2)) to obtain data on acceleration, time, and deformation. However, this would be an approximation that overlooks the complexity of this phenomenon, neglecting factors such as crushing, folding, buckling, and frictional contact between elements.

(2) $$F\cdot t=m\nu_{0}$$

The initial trajectory of forces during a frontal impact is highly relevant, as deceleration peaks over time have a direct correlation with occupant injuries. In contrast, the duration of the impact has a lesser influence.

In collisions, the kinetic energy transformed during the milliseconds of the impact pulse is distributed to the user primarily through two paths. The first path involves motion restriction systems, which include seat belts, airbags, and headrests. The second path is via programmed deformation zones (crumple zones), which can absorb more than 60% of the total energy.

The energy absorbed EA by these programmed deformation elements is governed by Equation (3), where *d* represents the deformation of the element itself.

(3) $$EA=\int_{0}^{d}F\left(x\right)\cdot dx$$

where *x* is the displacement, and F(x) is the crushing force.

Another key measurement parameter is the Specific Energy Absorption (SEA), which quantifies the amount of energy absorbed per unit mass. This metric is particularly important in the design and evaluation of mechanical energy absorbers, as it directly correlates with their efficiency and performance. Mechanical energy absorbers dissipate kinetic energy primarily through material deformation, converting the energy from impacts into plastic deformation within their structural components. Thin-walled structures are commonly employed in such absorbers due to their ability to achieve high SEA values while maintaining a lightweight design (Equation (4)). These structures are engineered to deform progressively under loading conditions, ensuring controlled energy dissipation and minimizing the severity of impacts.

(4) $$SEA=\frac{EA}{m}$$

The Mean Crushing Force (MCF) is a critical parameter for evaluating the efficiency of impact pulses. This measurement is designed to ensure uniformity in the behavior of energy absorbers across the operational range. MCF represents the average force exerted during the crushing process, providing insights into how consistently the absorber dissipates energy under dynamic loading conditions.

Mechanical energy absorbers rely on controlled deformation to dissipate kinetic energy effectively, and the MCF plays a key role in determining their performance. A higher MCF value typically indicates a more stable and predictable energy absorption profile, which is essential for applications where safety and reliability are paramount, such as crashworthiness in automotive structures.

(5) $$MCF=\frac{EA}{d}$$

In the crushing zone, key components can be identified, such as the bumper beam, crash box, and the side members that connect this structure to the chassis. Each of deformation elements plays a specific role in ensuring the efficiency and effectiveness of the energy absorption system:

- Bumper Beam: This component is designed to absorb initial impact forces and distribute them across the deformation zone. It acts as the first line of defense, reducing the severity of the impact before it reaches the more critical structural components.
- Crash Box: Positioned between the bumper beam and the side members, the crash box is engineered to deform plastically under impact, dissipating kinetic energy while preventing excessive force transmission to the main structure. Its modular design allows for easy replacement after a collision, making it a cost-effective solution for energy absorption.
- Side Members: These longitudinal beams provide structural support and guide the force flow during an impact. Their role is crucial in maintaining the integrity of the chassis and ensuring that the deformation zone performs uniformly across the operational range.

Together, these elements form a cohesive system that prioritizes occupant safety while minimizing damage to the vehicle’s core structure. The design and arrangement of these components are optimized to balance energy absorption, weight reduction, and structural reliability, making them essential in crashworthiness engineering for automotive and transportation industries.

To mitigate the severity of impacts, absorber elements are employed to dissipate kinetic energy. While energy dissipation can occur through fluid motion or heat transfer, mechanical absorbers achieve this by undergoing deformation. These absorbers are predominantly manufactured using thin-walled structures, as they offer the dual advantage of being lightweight and providing rigidity along the impact direction. The use of lightweight components has gained significant attention in modern product design due to their sustainable benefits, including reduced material consumption, lower energy demands, and minimized greenhouse gas emissions, all while delivering exceptional mechanical performance [30].

The strategic placement of the Crash Box is a key aspect of its design. It is located along the force distribution path between the front bumper beam and the frontal rails of the chassis, which outlines the deformation zone. This placement ensures that the Crash Box acts as the primary energy absorbing element during a frontal collision, protecting critical structural components and minimizing damage to the vehicle´s core frame.

**Figure 3:**
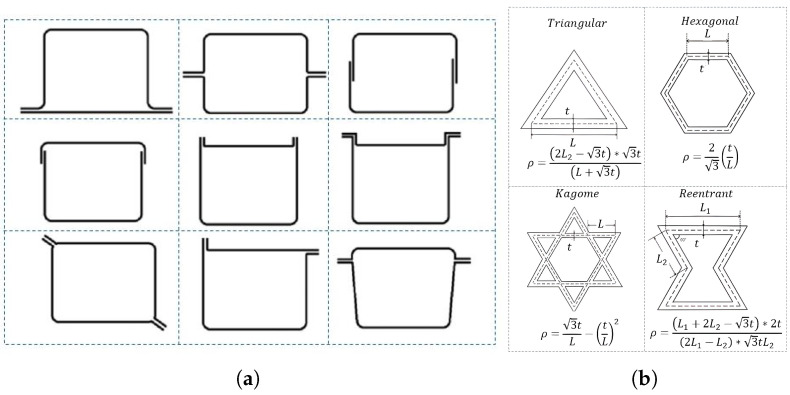
Honeycombs characteristics: (**a**) Crush section and (**b**) internal patterns.

The common use of sectional profiles joined by spot welding, as illustrated in Figure 3a, is driven by their proven behavior under impact scenarios and their manufacturability. These profiles are widely applied in automotive engineering due to their ability to provide structural stability during deformation while being cost-effective and feasible for large-scale production.

## 3. Experimental Methodology

The passive safety area seeks to reduce the severity of impacts for both occupants and pedestrians. There are different indicators to know the probability of biomechanical damage, which is the result of both the amplitude and the duration of the impact. To dissipate the impact energy, elements that transform kinetic energy into deformation energy are used. In order to evaluate the feasibility of using printed thin-wall elements to perform this function of mechanical absorbers, customization of the printing parameters will be carried out, their behavior in crushing will be evaluated experimentally. To improve the Peak Crushing Behavior with a customization thickness.

### 3.1. Materials

Polylactic acid (PLA) is a versatile and biodegradable biopolymer. Unlike petroleum-based polymers, PLA is derived from renewable raw materials such as corn, sugarcane, and tapioca [31]. Furthermore, it is recyclable and compostable under industrial conditions, making it a more sustainable and environmentally friendly alternative. These features have led to increased adoption across various industries in recent years [32].

The thermal properties of polylactic acid (PLA) are typically characterized using differential scanning calorimetry, thermogravimetric analysis, and dynamic mechanical analysis, the main characteristics are summarized in Table 1. These properties are directly influenced by factors such as thermal history, polymerization conditions, molecular weight, among others. In the thermal analysis of polymers, various studies and reference values are particularly relevant for PLA, including its melting temperature $\left(T_{m}\right)$ within the range of 150–162 °C and its glass transition temperature $\left(T_{g}\right)$ between 45–60 °C. Prolonged exposure of PLA to specific conditions at temperatures above 200 °C may lead to minor material degradation, while complete degradation occurs within the range of 290–380 °C [33].

3D printing using polylactic acid (PLA), it is essential to adjust thermal parameters according to the material’s properties. The extrusion temperature typically ranges from 190 °C to 220 °C, ensuring proper melting and flow of the filament, while the bed temperature is set between 50 °C and 70 °C to enhance adhesion and reduce warping. Although PLA does not require a heated chamber, maintaining a controlled ambient temperature (20 °C to 40 °C) prevents issues like layer separation or uneven cooling. Factors such as filament quality, printing speed, and environmental conditions should also be considered. Proper calibration of the printer, including bed leveling and surface preparation, combined with moderate printing speeds (40–60 mm/s), ensures high-quality results with minimal defects.

When working with polylactic acid (PLA), the extrusion temperature must exceed the melting point ($T_{m}$) which ranges between 150 °C and 180 °C depending on the type of isomer—and achieve a balance between flowability and thermal stability. According to the study by [34], the optimal extrusion temperature ensures a balance between flexural and tensile modulus, promoting strong interlayer adhesion while avoiding thermal degradation. Additionally, the bed temperature plays a crucial role in maintaining the piece’s adhesion to the printer during manufacturing, minimizing detachment risks and preventing warping caused by rapid material contraction due to abrupt temperature changes. Based on recommendations from the material provider ColorPlus3D, the suggested bed temperature range is 20 °C to 60 °C. However, studies like Spoerk et al. [35] narrow the optimal range to 50–60 °C, considering the relationship between interfacial tension and adhesion forces. For practical purposes, a mid-range bed temperature of 5 °C is applied to ensure stability and adhesion during the printing process.

### 3.2. 3D Printing Setup

The test components were manufactured using a commercial 3D printing system, specifically the Bambu X1 Carbon model. The fabrication process was conducted at a temperature of 200 °C within the liquefier chamber and 50 °C on the build platform. The key characteristics of the printed components are detailed in Table 2.

**Figure 4:**
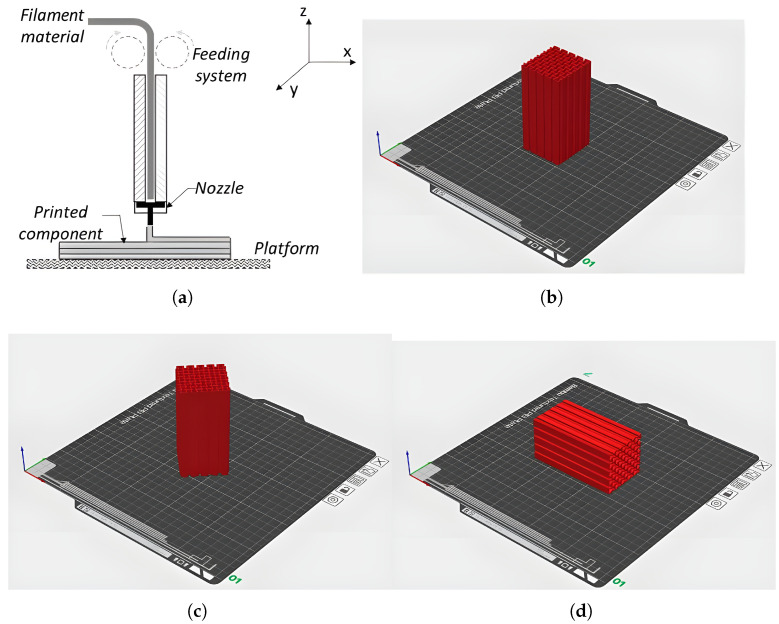
Schematic manufacturing process: (**a**) additive process (**b**) printer configuration at raster 0° and plane XZ, (**c**) printer configuration ar raster at 45° and (**d**) direction plane XY.

Considering the variation of parameters that can be studied in additive manufacturing, this analysis focuses on raster angle, print direction, and the influence of geometry type on energy absorption. The selected parameters are applied to devices subjected to heat treatments aimed at improvement. By definition, the raster angle refers to the material deposition angle during the layer-by-layer construction process in additive manufacturing, typically measured relative to a reference axis, commonly the X-axis of the printer. Figure (Figure 4b) shows the re-entrant honeycomb with a raster of 0°, and (Figure 4c) shows the same design with a raster at 45°. The manufacturing direction, defined by the printing position relative to the plane of the energy absorption device, significantly impacts mechanical properties and energy absorption performance, necessitating comparative evaluations across different unicellular geometries (Figure 4d).

In the context of material extrusion printing, the controlled environment provided by a closed chamber offers advantages regarding the drying speed of the material, as it minimizes the temperature gradient with the surrounding environment. The relationship between ambient conditions and PLA crystallization was thoroughly analyzed by [36], exploring a spectrum of ambient temperature ranges between 40 °C and 70 °C. Based on the detailed findings of this study, it can be inferred that printing PLA at 40 °C promotes molecular entanglement without inducing accelerated crystallization, which could lead to internal stresses. This ensures consistent mechanical properties in the printed parts.

Annealing is a thermal process that involves heating the printed component to a temperature within the range of the polymer’s glass transition and melting point, followed by gradual cooling. For semi-crystalline polymers such as PLA, this procedure enhances the crystalline phase content and promotes diffusion and re-bonding of polymer chains at layer interfaces, thereby improving the mechanical integrity of the part. The annealing process was conducted using a Thermolyne FA1740-1 furnace (Dubuque Iowa, MS, USA), equipped with a programmable temperature controller and calibrated thermocouples to ensure precise temperature regulation. Prior to treatment, the furnace was preheated and maintained at the target temperature for two hours to achieve uniform thermal conditions. After the annealing duration, the furnace was turned off, allowing the component to cool naturally inside. This standardized cooling protocol was consistently applied to all samples unless specified otherwise [27]. This approach minimizes warpage by maintaining dimensional stability within the specified temperature range [37].

### 3.3. Mechanical Testing

**Figure 5:**
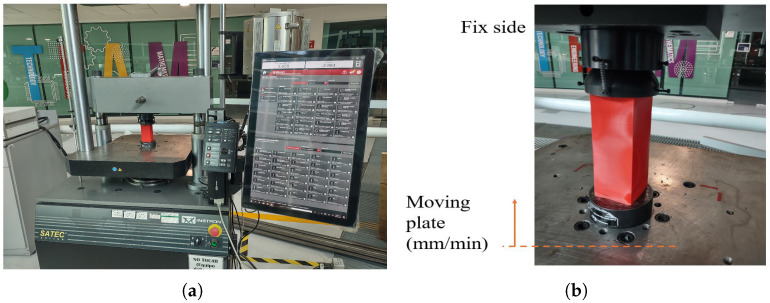
Experimental crushing test set up: (**a**) Universal test machine and (**b**) detail in compression plates.

To assess the performance of an energy absorber, a crushing test was conducted on components in two conditions: as-printed (Raw) and after undergoing post-thermal treatment. This evaluation aimed to compare the reference state of the material with the effects of the thermal treatment. The experimental tests were carried out using an Instron universal testing machine equipped with a Blue Hill controller, ensuring precise control and data acquisition. Measurements were recorded at a sampling frequency of 0.2 s to capture detailed force-displacement behavior during the test. The experimental setup involved fixing one side of the component securely, while the other side was subjected to vertical movement to apply the crushing force (Figure 5). The test was performed at a controlled rate of 10 mm/min to ensure consistency and minimize dynamic effects. This methodology allowed the characterization of the mechanical response under compressive loading conditions.

## 4. Results and Discussion

**Figure 6:**
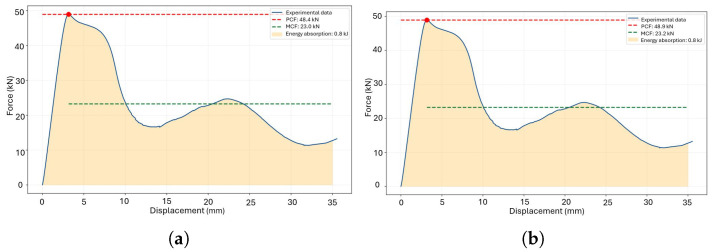
Crushing force in the raw printed thin-walled crash box Reentrant Honeycomb: (**a**) raster 0° and (**b**) raster 45°.

The resulting energy absorption is illustrated in Figure 4. To evaluate the influence of raster angle under out-of-plane loading, a comparison is made between a 0° angle (Figure 6a) and a 45° angle (Figure 6b), both featuring a re-entrant honeycomb cross-section and a specific weight.

The energy absorption process in structures during crushing is critical due to its stable behavior throughout the test, as evidenced by the deformation. This stability is reflected in the energy absorption metrics, including Specific Peak Crushing Force (SPCF), Specific Mean Crushing Force (SMCF), and Specific Energy Absorption (SEA), summarized in Table 3.

**Figure 7:**
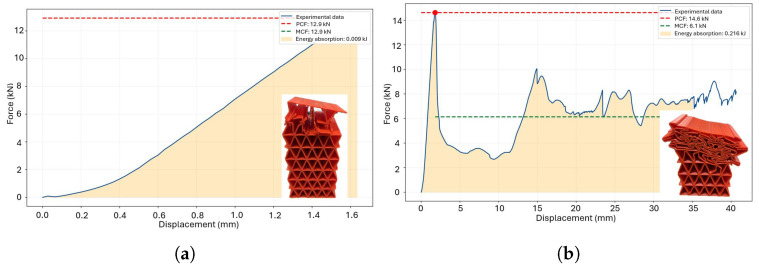
Manufacturing plane direction: (**a**) Direction XY and (**b**) Direction XZ.

Tests reveal that manufacturing in the XY plane results in excessive rigidity due to the layer orientation, leading to premature, sectional failures as observed in Figure 7a, making it unsuitable for the proposed design. Conversely, manufacturing in the XZ plane ensures structural integrity throughout the test (Figure 7b) but exhibits energy absorption instability, marked by sudden peaks caused by structural re-accommodation whenever individual elements fail. Additionally, variations in the printing path, driven by changes in cross-sectional geometry, further influence mechanical behavior and energy absorption. Therefore, optimizing the manufacturing direction and printing path is critical to achieving a balance between structural integrity and stable energy absorption.

The next parameters analyzed involves an inverted triangular honeycomb geometry, with its cross-section. This unicellular structure features periodic reinforcement along the Y-axis, designed to achieve a uniform force distribution and evenly support loads during energy absorption. However, experimental results reveal a short deformation range before total failure, indicating fragility in this geometry for both tested scenarios. It is concluded that the sharp angles characteristic of this design lead to early stress concentration, which accelerates failure. Table 4 summarizes the metrics. While the unicellular distribution of the inverted triangular honeycomb geometry is inherently fragile, a notable 42% difference in SPCF (Specific Crush Force Efficiency) values is observed. This variation is attributed to the manufacturing direction, which significantly influences the structural behavior and energy absorption capacity of the geometry.

**Figure 8:**
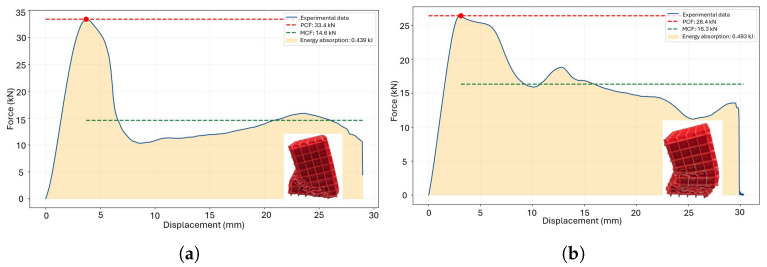
Crushing force in the raw printed thin-walled crash box Cubicfoam: (**a**) plane XY and (**b**) plane XZ.

The cubicfoam, a square honeycomb geometry with orthogonal reinforcements forming cubic subdivisions, creates a three-dimensional network of interconnected cubes. Under load, forces are distributed across walls, edges, and primarily at the interconnection nodes. Comparative evaluations under different manufacturing directions, as shown in Figure 8a and Figure 8b, reveal stable behavior during the initial deformation range (up to 27 mm and 29 mm, respectively). However, stress concentration at the weakest element triggers localized collapse, leading to crack propagation throughout the structure and a significant loss of energy absorption capacity beyond this point. Optimizing manufacturing direction and reinforcement design could enhance the structural performance and energy absorption efficiency of the cubicfoam geometry.

According to the results presented in Table 5, manufacturing in the XY plane demonstrates a 33% higher SPCF compared to the XZ plane, highlighting the variation in stiffness due to manufacturing orientation. However, in terms of SMCF and SEA, the XZ plane exhibits superior performance, as the plateau region maintains a higher average force throughout the displacement process.

It is concluded that this geometry can be utilized in various energy absorption applications, considering its limitations regarding the ultimate stress in this type of geometric arrangement. For optimal performance, an orthogonal printing plane relative to the load, as evaluated in this study, should be used.

The hexagonal honeycomb with transverse geometry in the XZ plane, represents a classic case study of single-cell geometries. Its use in energy absorption applications has been proposed for decades. However, it is essential to analyze the additive manufacturing process in relation to the printing plane and its behavior, which is influenced by inherent mechanical properties.

**Figure 9:**
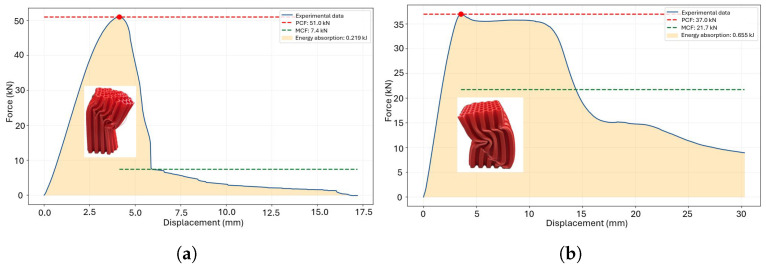
Crushing force in the hexahonal diamond honeycomb: (**a**) plane XY and (**b**) plane XZ.

As with previous cases, the stiffness of the element, affected by the constructive nature of the device, is reflected in the disparity of SPCF values. For the device printed in the XY plane (Figure 9a), failure occurs after surpassing the elastic zone, resulting in the absence of an energy absorption mechanism. In contrast, the device manufactured in the XZ plane (Figure 9b) remains stable throughout the entire loading process, demonstrating its viability for application according to the SMCF and SEA values described in Table 6 for the proposed design.

From the experimental evaluation with variations in manufacturing direction, it can be concluded that, despite differences in transverse geometry direction, single-cell geometry type, and post-load deformations of the device, energy absorption performance is significantly higher in the XZ plane. The root cause of this lies in the influence of the constructive nature of additive manufacturing on energy absorption, where layer adhesion plays a fundamental role. Therefore, for the proposed device, the default manufacturing direction is established as the XZ plane.

### 4.1. Crushing Response Based on the Load

The geometric analysis of energy absorption devices based on the direction of external loading is categorized into in-plane and out-of-plane loads. In the case under evaluation, with the load applied along the Y-axis, an in-plane load occurs when the unicellular geometry is oriented within the XY plane, whereas an out-of-plane load corresponds to the geometry’s cross-section being positioned in the XZ plane. For out-of-plane loading, the energy absorption behavior under crushing was assessed for various geometries, including re-entrant honeycomb, cubic foam, triangular honeycomb, and inverse triangular honeycomb. The results indicate that, despite extensive research and continuous improvement in these configurations, their application for energy absorption in PLA under significant deformation is not feasible due to the occurrence of sudden or unstable failures during evaluation.

**Figure 10:**
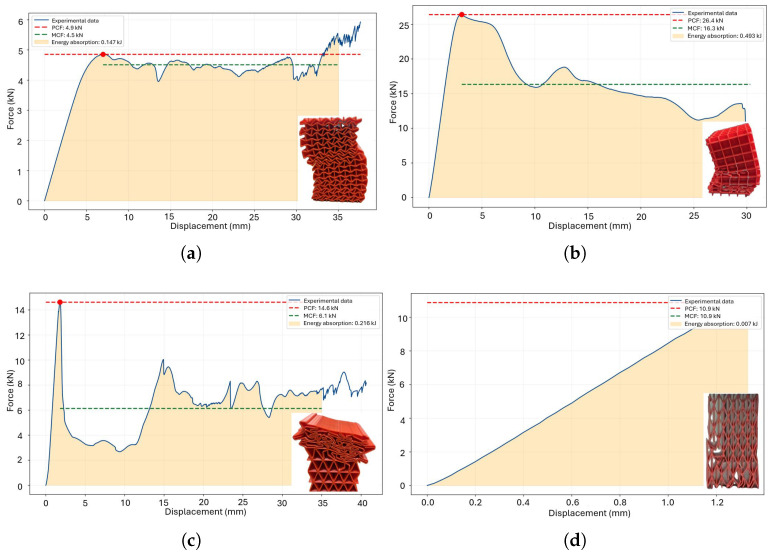
Crushing response with load in plane: (**a**) reentrant, (**b**) cubicfoam, (**c**) triangular and (**d**) inverted triangular.

The reentrant honeycomb structure exhibits low and unstable energy absorption under load due to insufficient in-plane stiffness, reaching a densification zone at approximately 30 mm of deformation. The cubicfoam unicellular geometry, which combines two square honeycomb planes, demonstrates good performance throughout the evaluation process but is ultimately unsuitable for the proposed application due to sudden failure and loss of load resistance at the end of testing, where stability is required for consistent energy absorption. Triangular honeycomb structures show uneven force distribution across specific elements, leading to instability. For instance, the triangular honeycomb (Figure 10c) experiences cascading failure, compromising energy absorption stability, while the inverse triangular honeycomb (Figure 10d) undergoes multiple sudden failures due to excessive stiffness.

The mass and specific energy absorption values are summarized in Table 7, respectively. Based on the specific energy absorption (SEA) data, only the cubicfoam geometry demonstrates outstanding performance. However, as previously mentioned, it is ultimately ruled out due to its sudden failure at the end of the test.

**Figure 11:**
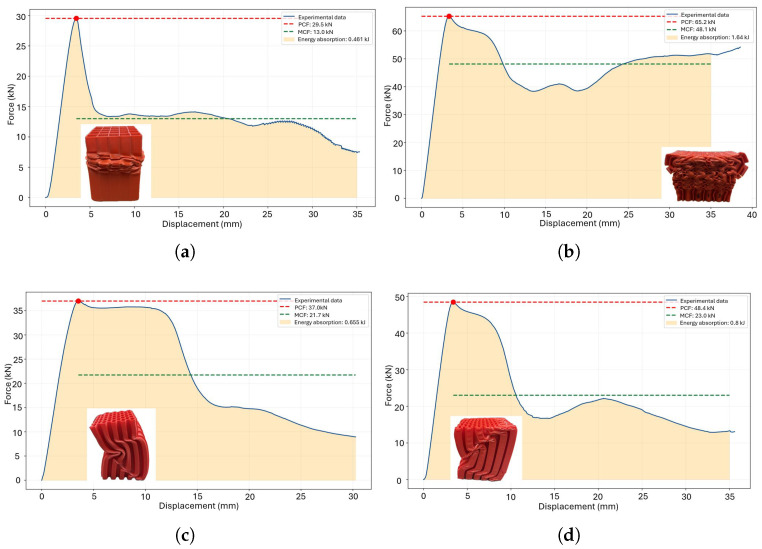
Crushing response with load out of plane: (**a**) reentrant, (**b**) cubicfoam, (**c**) triangular and (**d**) inverted triangular.

Out-of-plane geometries, including square (Figure 11a), triangular (Figure 11b), hexagonal (Figure 11c), and re-entrant (Figure 11d) honeycombs, were tested. According to the theory developed by Zhang and Ashby [38], these geometries are optimized for energy absorption; however, manufacturing methods at the time posed significant limitations. Today, additive manufacturing presents a promising opportunity to explore the feasibility of diverse energy absorption solutions. The post-load behavior of the evaluated geometries can be categorized into two types: square and triangular honeycombs exhibit progressive folding, which is the desired behavior for energy absorption devices. Conversely, re-entrant and hexagonal geometries demonstrate localized buckling in a “C” shape, typical of thin-walled tubes under axial loading.

The mass and specific energy absorption values are summarized in Table 8. Considering the load plane, post-load failure behavior, comparative mass differences, and energy absorption, the triangular and square out-of-plane honeycomb geometries were selected for thermal treatments aimed at improving energy absorption. Both devices demonstrate good energy absorption performance, ideal deformation folding, and significant mass differences, which enable an appropriate evaluation of the influence of thermal treatments on energy absorption devices.

### 4.2. Thermal Treatment

After selecting the geometric parameters through laboratory tests, the proposed thermal treatments are carried out to study their effect on energy absorption in the selected devices. It is important to note that a potential improvement in energy absorption behavior is anticipated due to the semicrystalline nature of PLA and the microstructural reorganization that influences its mechanical properties.

Annealing thermal treatments were performed on the selected energy absorption devices, specifically the triangular and square honeycomb geometries. These treatments were conducted at temperatures of 55 °C, 60 °C, 65 °C, and 70 °C, aiming to find the optimal combination of temperature and time. To achieve this, oven exposure times ranging from 1.5 h to 16 h were analyzed.

**Figure 12:**
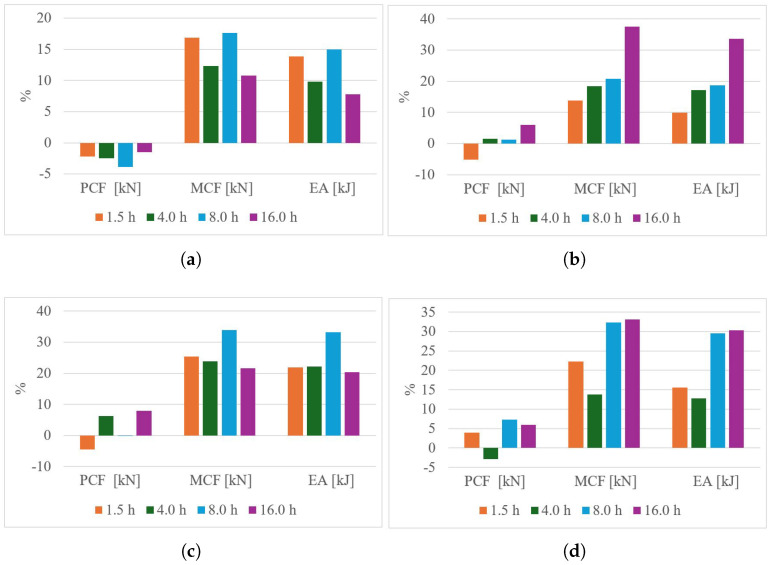
Crushing metrics for square honeycomb components subjected to thermal treatment: (**a**) 55 °C, (**b**) 60 °C, (**c**) 65 °C and (**d**) 70 °C.

**Figure 13:**
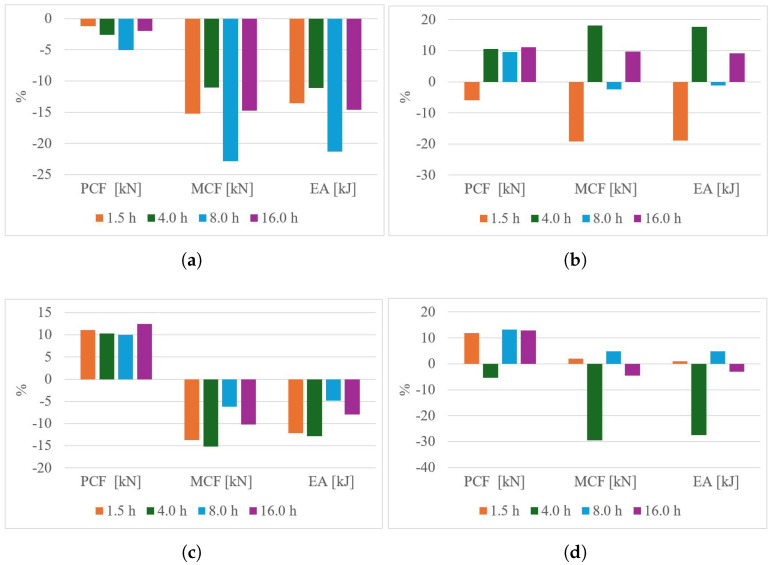
Crushing metrics for triangular honeycomb components subjected to thermal treatment: (**a**) 55 °C, (**b**) 60 °C, (**c**) 65 °C and (**d**) 70 °C.

The energy absorption requirements depend on the customization of the deformation process. The primary crushing force (PCF) acts as the main trigger for the deformation process; however, it must be combined with the mean crushing force (MCF) to effectively control and manage the deformation behavior. A combination of high MCF and low PCF results in ductile failure, as illustrated in Figure 12a. Additionally, a transition effect influenced by the treatment duration is observed at temperatures of 60 °C and 65 °C, corresponding to Figure 12b and Figure 12c, respectively. The optimal crushing performance is achieved with a thermal treatment at 70 °C, except for the case of a 4-h treatment duration (Figure 12d). Conversely, a high PCF combined with low MCF leads to brittle failure, as demonstrated in the triangular honeycomb structure subjected to thermal treatment for 1.5 h (Figure 13d).

The geometry plays a significant role in the performance under thermal treatment conditions. In the case of triangular honeycomb structures, the post−treatment process at 55 °C proved to be detrimental (Figure 13a–c). Overall, thermal treatment tends to induce a brittle behavior. While the optimal PCF is achieved at 60°C, it negatively impacts the MCF (Figure 13c).

After completing the experimentation process, it is essential to analyze how different thermal treatments influence energy absorption performance, starting from the hypothesis that such treatments improve energy absorption efficiency. The results show contrasting behaviors between the evaluated devices. For square honeycomb structures, energy absorption improves by up to 33% with treatments at 60 °C for 16 h and 65 °C for 8 h. Conversely, triangular honeycomb structures exhibit a decrease in energy absorption, with rare exceptions under specific thermal treatments. This highlights the impact of thermal treatments on PLA-based additive manufacturing devices. Furthermore, at 60 °C, 65 °C, and 70 °C, the peak crushing force (PCF) increases, theoretically attributed to thermal gradients affecting external walls due to the unicellular wall distribution.

## 5. Conclusions

This study focuses on optimizing the crushing performance of mechanical energy absorbers fabricated through additive manufacturing using PLA. The results highlight that 3D-printed PLA components not only offer design flexibility and customization but can also benefit significantly from thermal post-processing treatments to enhance their mechanical properties. By tailoring both the geometric design and material characteristics through controlled thermal treatments, it is possible to achieve lightweight, high-performance structures capable of efficiently absorbing energy during low-speed impacts. These findings underscore the potential of combining additive manufacturing with post-processing techniques to address specific engineering challenges, paving the way for innovative solutions in industries such as automotive, and protective equipment. Moreover, the study emphasizes the importance of understanding the interplay between material behavior, design parameters, and processing conditions, offering valuable insights for future research and development in advanced manufacturing technologies.

The 33% improvement observed in square honeycomb structures is attributed to different mechanisms: at 60 °C, the enhancement results from a combined increase in PCF and mean crushing force (MCF), while at 65 °C, the improvement is solely due to MCF. From a design perspective, the treatment at 60 °C for 8 h is preferable, as it balances peak and average crushing forces across various impact scenarios. At higher temperatures, such as 70 °C, structural instability likely arises from surpassing the glass transition zone, destabilizing the microstructure. In contrast, treatments at 55 °C fail to eliminate internal stresses and voids between layers, leading to no performance improvements, particularly in triangular honeycomb structures.

The study highlights several limitations that warrant further investigation. Future research should prioritize statistical analysis, to account for experimental variability and ensure reliable comparisons. Expanding testing to include multidirectional loading and conducting microstructural analyses with DSC, DMA and SEM can provide deeper insights into the effects of thermal treatments on void elimination, stress relaxation, and crystallinity.

## Figures and Tables

**Table 1:** Physical and mechanical properties of PLA.

Property	PLA
**Color**	red
**Density** (g/cm^3^)	1.24
**Content**	1000 g (100% PLA)
**Young’s Modulus** (MPa)	3.5
**Tensile Strength** (MPa)	60
**Shear Modulus** (MPa)	1287
**Poisson’s ration**	0.36
**Filament diameter** (mm)	1.75
**Extruder temperature** (°C)	180–230
**Bed temperature** (°C)	20–60

**Table 2:** Printing parameters for PLA specimens.

Printing Parameter	PLA
**Nozzle diameter** (mm)	0.4
**Layer thickness** (mm)	0.1
**Extruder temperature** (°C)	200
**Bed temperature** (°C)	50
**Printing rate** (mm/min)	50
**Build direction**	Flat, printed through the thickness
**Raster angle**	+/−45 °C

**Table 3:** Energy Absorption in Re-Entrant Honeycomb Structures with Raster Angle Variation.

Crushing Metric	Raster 0°	Raster 45°
**SPCF** (kN/kg)	508.39	513.43
**SMCF** (kN/kg)	241.66	243.86
**SEA** (kJ/kg)	8.39	8.39

**Table 4:** Energy Absorption in triangle Honeycomb Structures with Raster plane variation.

Crushing Metric	Plane XY	Plane XZ	Inverted	Inverted
			Plane XY	Plane XZ
**SPCF** (kN/kg)	159.24	184.60	94.21	133.87
**SMCF** (kN/kg)	159.24	77.40	80.09	133.87
**SEA** (kJ/kg)	0.11	2.72	0.046	0.09

**Table 5:** Energy Absorption in cubicfoam Honeycomb Structures with printing plane variation.

Crushing Metric	Plane XY	Plane XZ
**SPCF** (kN/kg)	492.98	329.98
**SMCF** (kN/kg)	184.68	204.12
**SEA** (kJ/kg)	5.553	6.16

**Table 6:** Energy Absorption in Honeycomb Structures with printing plane variation.

Crushing Metric	Plane XY	Plane XZ
**SPCF** (kN/kg)	566.713	410.99
**SMCF** (kN/kg)	82.396	241.44
**SEA** (kJ/kg)	2.437	7.28

**Table 7:** Energy Absorption in Structures with load in plane.

Crushing Metric	Reentrant	Cubicfoam	Triangular	Triangular Invert
**Mass** (g)	96	80	81	109
**SPCF** (kN/kg)	50.54	329.98	184.6	133.87
**SMCF** (kN/kg)	49.92	204.12	77.4	133.87
**SEA** (kJ/kg)	1.53	6.16	2.72	0.09

**Table 8:** Energy Absorption in Structures with load out of plane.

Crushing Metric	Square	Triangular	Hexagonal	Reentrant
**Mass** (g)	60	125	90	95
**SPCF** (kN/kg)	462.26	521.901	410.99	508.39
**SMCF** (kN/kg)	216.4	385.093	241.44	241.66
**SEA** (kJ/kg)	7.68	13.147	7.276	8.36

## Data Availability

The original contributions presented in this study are included in the article. Further inquiries can be directed to the corresponding author.
